# First Phenotypic Characterization of the Edible Fruits of *Lardizabala biternata*: A Baseline for Conservation and Domestication of a Neglected and Endemic Vine

**DOI:** 10.3390/plants14203126

**Published:** 2025-10-10

**Authors:** Jaime Herrera, Leonardo D. Fernández

**Affiliations:** 1 Graduate School, Faculty of Agricultural Sciences, Campus Isla Teja, Universidad Austral de Chile, Valdivia 5090000, Chile; herrera.p.jaime@gmail.com; 2 Centro de Investigación en Recursos Naturales y Sustentabilidad (CIRENYS), Universidad Bernardo O’Higgins, Santiago 8320000, Chile; 3 Graduate School of Agronomy, Núcleo de Investigación en Sustentabilidad Agroambiental (NISUA), Facultad de Medicina Veterinaria y Agronomía, Universidad de Las Américas, Manuel Montt 948, Providencia, Santiago 8320000, Chile

**Keywords:** biodiversity hotspot, domestication, Lardizabalaceae, Lardizabaloideae, fruit size, fruit weight, morphological traits, seed number, potentially endangered plant, edible fruits

## Abstract

*Lardizabala biternata* is a culturally valued, endemic vine of the Chilean Winter Rainfall–Valdivian Forest biodiversity hotspot, traditionally harvested for its sweet, edible fruits. Despite its ecological singularity as the sole species in a monotypic genus, the species remains biologically and agronomically understudied, with no formal cultivation systems. There is currently no baseline information on its fruit morphology, which limits the design of conservation strategies and the development of its agronomic potential. This study provides the first phenotypic characterisation of *L. biternata* fruits, aimed at supporting germplasm evaluation, ex situ conservation, and sustainable domestication of this rare species. A total of 205 fruits were sampled across two seasons and two geographically distant populations. We measured 14 traits, including weight, length, diameter, pulp content, and seed metrics, and analysed morphological variation using *t*-tests, ANOVA, regression, and principal component analysis or PCA. Fruits averaged 21.0 g in weight, 54.2 mm in length, and 23.8 mm in diameter. Edible pulp constituted 44.4% of total fruit weight and showed strong positive correlations with fruit size, seed number, and seed weight. Significant differences were observed across seasons and populations, with cooler, wetter conditions associated with larger fruits and higher pulp yield. Our findings reveal substantial morphological variability and climate sensitivity, providing a crucial baseline for selecting desirable traits. This work informs ongoing efforts in plant domestication, sustainable agriculture, and the conservation of underutilised species of cultural and ecological importance.

## 1. Introduction

**Figure 1:**
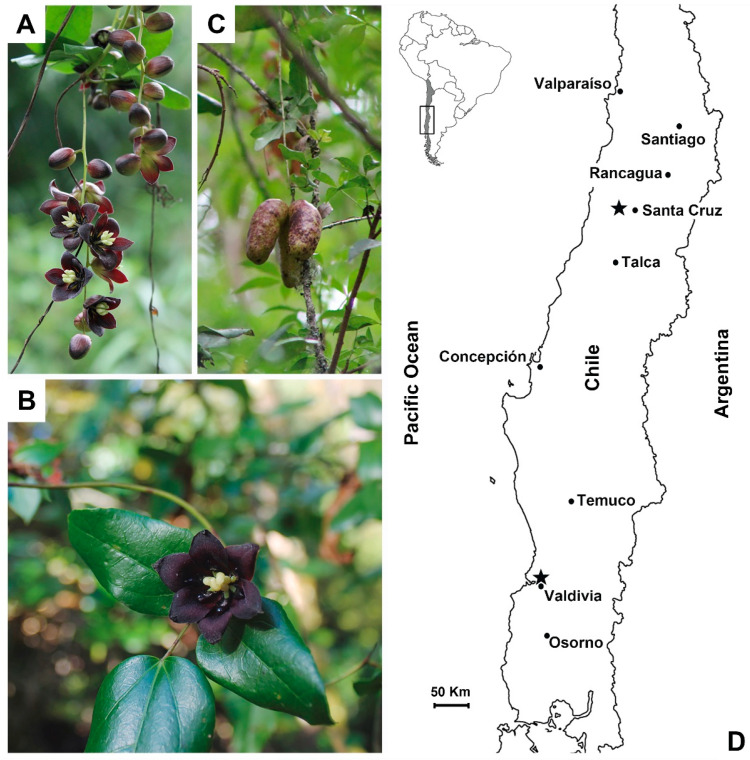
Flowers, fruits, and distribution of *Lardizabala biternata* in central Chile. (**A**) Male flowers, (**B**) female flower, (**C**) fruits, and (**D**) sampling location of *L. biternata* fruits. Partial map of central Chile showing the locations of fruit collection near Santa Cruz (2016 season) and Valdivia (2016 and 2018 seasons), indicated by black stars. The inset map displays South America, with a highlighted box indicating the study area.

*Lardizabala* [1] is a monotypic genus in the family Lardizabalaceae. Its only species, *Lardizabala biternata* Ruiz & Pav., is a climbing plant endemic to Chile (Figure 1A–C). Among native communities, the plant is traditionally known as Nüpu-foki, and its fruits as *Kówell* or *Cóguil* [2]. The species is currently known by various vernacular names, including *coguilera*, *boqui-cóguil*, *coile*, *coguilvoqui*, *collivoqui*, and *cógüil* [3,4], while the fruits are commonly called *Cóguil* or *Coile* [5]. Sometimes, the same name is used for both the plant and its fruits [6].

*L. biternata* occurs within the Chilean Winter Rainfall–Valdivian Forest biodiversity hotspot, a global centre of plant, animal and microbial endemism [7,8,9,10,11,12,13]. It inhabits evergreen forests across ~900 km of latitude (32–40° S) in central–southern Chile, from 100 to 1000 m above sea level [5,14] (Figure 1D). The species has been recorded in diverse ecological zones, including the pre-Andean region [15], coastal range [16], and several protected areas [17,18]. A record from Juan Fernández Island [19] remains unconfirmed. *L. biternata* is rare and sparsely distributed, with populations often fragmented and concealed within the understorey of native temperate forests [20]. This ecological habit renders it particularly difficult to locate and identify in the field, as it tends to blend with surrounding vegetation. Locating mature individuals and accessing fruiting specimens requires intensive sampling and specialised botanical expertise.

The species is identifiable by its flexible stems and dark green, coriaceous leaves, typically bi- or tri-ternate in arrangement [5,20,21]. It is dioecious [6]; male plants produce dark violet flowers in pendent clusters (Figure 1A), while female plants produce solitary flowers of similar colour (Figure 1B) [21]. The fruits are ovoid to oblong, approximately 5 cm along their longest axis (Figure 1C), with greenish to yellowish epicarp variably marked by violet spots [3,4]. Internally, they contain several black seeds embedded in a whitish, sweet-tasting pulp, which is locally consumed and appreciated [21].

Despite its longstanding cultural and gastronomic value, *L. biternata* remains largely understudied. No cultivation systems have been developed [14], and fruits are still harvested from the wild. Its nutritional, pharmacological, and functional properties remain virtually unexplored, with most available information drawn from historical texts [21,22,23], botanical compilations [3], or culinary and ethnobotanical guides [24,25,26]. To date, the only study that has directly focused on the species is that of Silva and Mancinelli, who extracted oleanolic acid from its leaves [27]. Other mentions of *L. biternata* appear only marginally in broader studies, such as those addressing wood anatomy [28] or molecular systematics in plants [29]. More recently, intact seeds found in native fox scats suggest a possible role for mesocarnivores in dispersal, though their effectiveness remains unclear [30].

A major knowledge gap concerns the species’ reproductive biology, particularly the phenotypic (morphologic and morphometric) diversity of its fruits. Fruit phenotype, defined as the physical and structural features of fruits, including shape, size, colour, texture, composition, and internal organisation, is widely used to identify cultivars and assess productivity [31,32,33,34,35,36,37,38]. In other Lardizabalaceae species, including *Decaisnea insignis*, *Akebia quinata*, and *Stauntonia obovatifoliola*, fruit size and weight correlate with yield and quality parameters [31,39,40,41]. Morphological traits also serve as proxies for plant development, environmental responses, and agronomic performance [31,42,43,44,45]. Their absence in *L. biternata* hinders informed decisions regarding conservation, variety selection, agronomic management, and yield optimisation [46,47,48,49,50,51,52].

This gap is particularly concerning for an endemic species distributed along a broad latitudinal gradient, subject to contrasting thermal and hydric regimes and likely sensitive to climate change [53,54,55,56]. In its native range precipitation increases and temperature decreases from north to south. The region has undergone substantial environmental change, driven by climatic shifts and anthropogenic pressures [53,54,55,56,57]. Although genotype plays a central role in determining morphology, phenotypic traits also respond to local environmental conditions, including temperature, precipitation, and seasonal variability [18,31,43,58]. The fruits of *L. biternata* may reflect both genetic differences and responses to environmental heterogeneity.

We hypothesise that (1) the morphological traits of *L. biternata* fruits differ significantly between two geographically distant and climatically contrasting populations, i.e., Santa Cruz (drier and warmer) and Valdivia (wetter and cooler), reflecting local environmental adaptation; and (2) fruit morphology varies between seasons because of interannual climatic variability. We expect differences in fruit weight, size, seed number, and trait correlations. To test these hypotheses, we pursued two goals: (i) to qualitatively and quantitatively describe the fruit morphology of *L. biternata* using criteria established for other members of Lardizabalaceae [31,39,41], and (ii) to assess correlations among key morphological traits. Our findings will establish a baseline for selecting desirable traits and contribute to understanding the species’ development, ecology, and cultivation potential.

## 2. Materials and Methods

### 2.1. Study Design and Sampling Sites

To evaluate phenotypic variation in *L. biternata* fruits, we collected samples from three population-year combinations: Santa Cruz 2016 (StCr16), Valdivia 2016 (Vald16), and Valdivia 2018 (Vald18) (Figure 1D). These sites represent climatically contrasting regions within the northern and southern boundaries of the species’ distribution range in Chile.

Santa Cruz (34°70′74.9″ S, 71°46′57.7″ W), located in central Chile, has a warm Mediterranean climate with summer maximum temperatures ranging from 27 to 31 °C and an average annual precipitation of approximately 700 mm, concentrated in the winter months [59]. Between 1980 and 2010, the average maximum temperature of the warmest month (January) was 30.1 °C, projected to rise to 32.4 °C by 2050 [60,61]. Valdivia (39°80′42.8″ S, 73°24′95.7″ W), ~500 km to the south, has a temperate oceanic climate with Mediterranean influence. During the same period, its average maximum summer temperature was 22.3 °C, and annual precipitation exceeded 2300 mm, also concentrated in winter [45,52]. By 2050, projections indicate temperatures >24 °C and reduced rainfall (<1592 mm) [60,61].

For climatic comparisons, we considered the period from March to February of the following year, aligning with traditional harvesting practices and the biological maturation of *L. biternata* fruits, as inferred from field observations and fruit fall patterns (Figure S1). This approach allowed us to capture the climatic conditions potentially influencing fruit development throughout the year.

### 2.2. Fruit Collection and Handling

**Figure 2:**
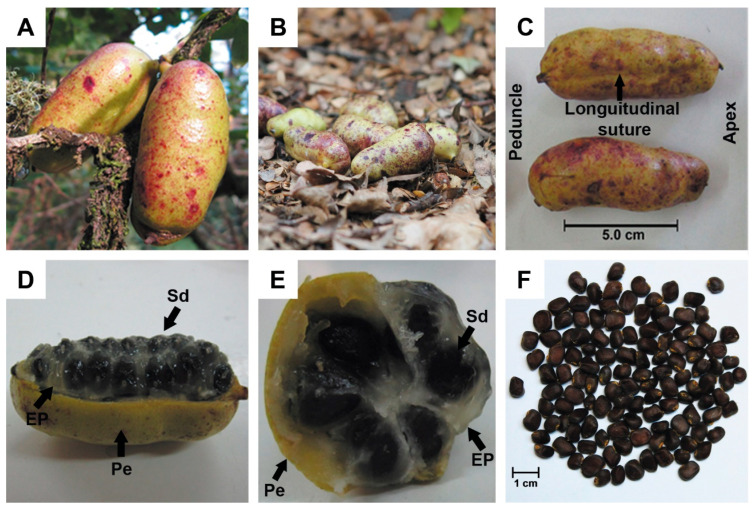
Fruits and seeds of *Lardizabala biternata*. (**A**) Fruits on the plant, (**B**) fruits on the soil, (**C**) fruits collected, (**D**) fruit dissected in frontal (**E**) and transversal planes, and (**F**) seeds of *L. biternata*. Letters indicate the edible pulp (EP), seeds (Sd) and peel (Pe) of *L. biternata* fruits. Bars represent scales of 5.0 and 1.0 cm.

In Santa Cruz, 53 ripe fruits were collected in late February 2016 (StCr16). In Valdivia, 73 fruits were collected in mid-March 2016 (Vald16), and 79 in mid-March 2018 (Vald18). Fruits were harvested randomly from climbing vines in the understorey or collected immediately after natural fall (Figure 2A,B). Samples were stored in sealed plastic bags with gel ice packs, transported to the laboratory on the same day, and processed promptly to minimise dehydration and weight loss. Due to the species’ growth form as a climbing liana and the lack of information on its clonal propagation (e.g., via rhizomes or stolons), it was not possible to attribute fruits to specific individual plants. Vines are often highly intertwined across multiple host trees, making it unclear whether different branches originate from the same or distinct genets. Therefore, all fruits from a given site and year were treated as a single population unit. For analytical purposes, the fruits from each of the three site-year combinations (StCr16, Vald16, Vald18) were treated as independent populations.

### 2.3. Morphological Characterisation

Each fruit was subjected to qualitative and quantitative morphological analysis. Qualitative variables included external and internal colouration and general shape. Quantitative traits included 14 morphological or morphometric variables distributed within three categories (i) weight and size of fruit: individual fresh weight (IFW), fruit length (FL), width (FW), height (FH), diameter (FD), and volume (FV); (ii) edible and structural components of fruits: edible pulp weight (EPW), peel weight (PeW), total seed weight (SdsW) and edible pulp plus peel weight [(EP + Pe)W]; and (iii) seed characteristics: total seed number per fruit (TSdn°), viable seeds (VSdn°), non-viable seeds (FSdn°), and average seed weight (ASdW). All fruits were measured for IFW, FL, FW, FH, FD, and FV. Subsamples were selected randomly for detailed dissection and component analysis [EPW, SdsW, PeW, (EP + Pe)W, TSdn°, VSdn°, FSdn° and ASdW], 15 fruits from StCr16, 20 from Vald16, and 10 from Vald18. Although the subsample represented only a fraction of the total number of fruits, the methodological and destructive design limited the analysis of additional morphological traits. The results were consistent with the statistical analyses applied, and the patterns observed across locations and years support the reliability of the findings.

**Figure 3:**
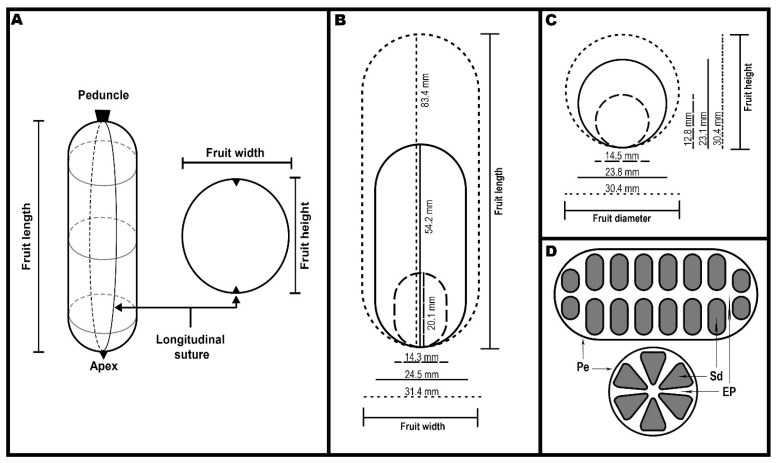
Morphological representation of *Lardizabala biternata* fruits. (**A**) General model showing fruit length, width, height, and longitudinal suture; (**B**) length and width of average (solid line), longer (dotted line), and shorter (dashed line) fruits; (**C**) height and diameter of average (solid line), longer (dotted line), and shorter (dashed line) fruits; (**D**) arrangement of seeds within the fruit under longitudinal and transverse sections, showing the edible pulp (EP), seeds (Sd), and peel (Pe).

Weights were recorded with an analytical balance (Mettler Toledo XP205DR, Greifensee, Switzerland), and dimensions were taken using a digital calliper (6-inch 150 mm Digital Calliper, China). FL was measured from the peduncle to the apex (Figure 2C and Figure 3A). FW and FH were measured relative to the longitudinal suture (Figure 2C and Figure 3A,B). FW was measured perpendicular to the suture, while FH was measured as the distance between the sutures on both sides of the fruit. FD was estimated as the average of FW and FH [FD = (FW × 0.5) + (FH × 0.5)].

*L. biternata* fruits are described as cylindrical-globose berries [3,4]; we therefore estimated fruit volume (FV) using a cylindrical geometric model: FV = FL × π × (FD × 0.5)^2^. This approach assumes regular cross-sections along the fruit’s longitudinal axis, consistent with field descriptions, our visual assessments, and the variation illustrated in Figure 3. We did not validate this model using water displacement in the present study but had previously applied and compared both methods in morphometric analyses of other plant structures (e.g., wheat grains), finding no significant differences between estimated and measured volumes [62]. The strong correlation observed between estimated volume and fresh weight supports the internal consistency and suitability of this model as a geometric proxy for fruit size in *L. biternata.*

To estimate structural components, peel was manually separated using a scalpel. The inner side of the peel was then gently scraped with a spoon to remove any remaining edible pulp, rinsed under running water, carefully dried using tissue paper and weighed in electronic balance. Seeds were place in a metal strainer, washed under running water, and gently pressed with a sponge to remove the adhering pulp and retain the seeds. Floating seeds were considered and counted as non-viable (FSdn°) (Figure S2), while those that settled were classified and counted as viable seeds (VSdn°). A triphenyl tetrazolium chloride (TTC) assay was conducted on a subset of floating and non-floating seeds to validate embryo viability. This histochemical test revealed no staining in floating seeds, supporting their classification as putatively non-viable. After air-drying, all viable seeds were weighed using an electronic balance. ASdW was estimated as the ratio of total seed dry weight to the number of viable seeds (ASdW = SdsW/VSdn°). EPW was calculated as EPW = IFW − (SdsW + PeW), and (EP + Pe)W was estimated as (EP + Pe)W = EPW + PeW. Component percentages were calculated relative to IFW.

### 2.4. Statistical Analysis

#### 2.4.1. Hypothesis 1: Spatial Variation (StCr16 vs. Vald16)

To evaluate differences in fruit morphology between the Santa Cruz and Valdivia locations during the same sampling year, Student’s *t*-tests were applied to compare the means of all fruit traits between StCr16 and Vald16. A pairwise regression analyses were conducted between traits, and comparison of regression lines (slope and intercept) was used to evaluate whether trait relationships differed between locations or sites.

#### 2.4.2. Hypothesis 2: Seasonal Variation (Vald16 vs. Vald18)

To assess year-to-year morphological variation within Valdivia, the same statistical approach was used to compare Vald16 and Vald18. Student’s *t*-tests examined differences in trait means, while regression comparison tested whether morphometric profiles and trait associations varied across years.

### 2.5. Supporting Analyses

For all populations, descriptive statistics, including the average (mean), maximum (max), minimum (min), standard deviation (SD), standard error (SE), and coefficient of variation (CV), were computed. Normality was assessed using the Shapiro–Wilk test (*n* < 50) or the Kolmogorov–Smirnov test (*n* > 50), depending on the sample size. Homoscedasticity was tested using Levene’s test prior to conducting parametric analyses. One-way ANOVA was used to assess intra-fruit variation in size and structure (e.g., FL vs. FW vs. FH, and EPW vs. PeW vs. SdsW). As the ANOVA test was performed intra-fruit and separately for each location or season, the groups did not differ in sample size. When significant differences were detected, post hoc comparisons were conducted using Tukey’s HSD test (α = 0.05). Students’ *t*-tests were also applied to compare mean trait values between the two populations sampled in the same season (StCr16 vs. Vald16), and between two seasons sampled in the same population (Vald16 vs. Val18). Although some groups differed in sample size due to logistical constraints, the framework remains robust under moderate heterogeneity, and has been successfully applied in similar fruit morphology studies with unbalanced samples (e.g., Zou et al., 2019) [44]. Therefore, we consider the approach appropriate for detecting general patterns in trait variation across site-years. Regression analyses were conducted to investigate trait correlations, stratified by location and season, using Statgraphics Centurion XVI test (StatPoint Technologies, Warrenton, VA, USA). Principal Component Analysis (PCA) was employed to visualise patterns of multivariate trait variation across populations and growing seasons. All statistical analyses were conducted using INFOSTAT [63] and Statgraphics, ensuring analytical consistency and reproducibility.

## 3. Results

### 3.1. Climatic Conditions at Study Sites

Temperature and rainfall profiles confirmed the climatic contrast between the two sampling locations (Table 1, Figure S1). In Santa Cruz (StCr16), located at the northern limit of *L. biternata*’s distribution, average minimum and maximum air temperatures were 8.8 °C and 23.1 °C, respectively, with occasional peaks of >30 °C during the summer. Annual precipitation between March 2015 and February 2016 was 586 mm, with most rainfall concentrated in winter and early spring.

In Valdivia (Vald16 and Vald18), temperatures were lower than in Santa Cruz, with average minimum and maximum values of 6.9 °C and 17.0 °C, respectively, and annual precipitation averaged 1994 mm (Table 1, Figure S1). These environmental differences support the spatial comparison of fruit traits.

### 3.2. General Fruit Characteristics

Qualitative observations showed that *L. biternata* fruits were mostly ellipsoidal or cylindrical (Figure 2A–C and Figure S3), with one end often broader than the other (Figure 3A–C). Mature fruits developed bumps and granules beneath the peel, giving a rough texture (Figure 2C). The peel changed from olive green (immature) to yellow, often covered with irregular purple spots (Figure 2A–C). A longitudinal section revealed white or pale grey pulp surrounding axially arranged black seeds (Figure 2D–F and Figure 3D). A cross section further revealed that the seeds exhibit axile placentation and are radially arranged, forming a circular pattern in the centre of the fruit (Figure 2E and Figure 3D).

### 3.3. Fruit Weight and Size

**Figure 4:**
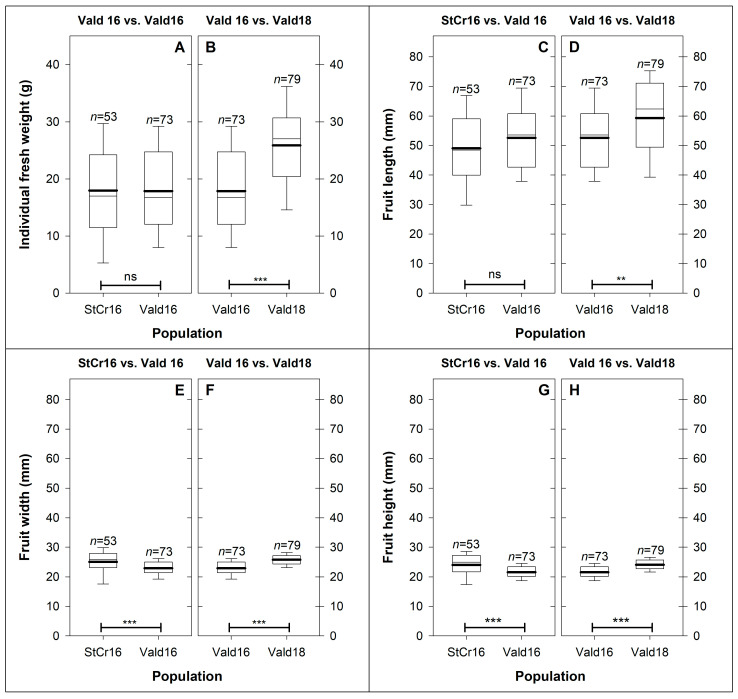
Weight and size of *Lardizabala biternata* fruits. (**A**,**B**) Individual fresh weight (g); (**C**,**D**) fruit length; (**E**,**F**) fruit width; and (**G**,**H**) fruit height of *L. biternata* fruits collected in Santa Cruz during the 2016 season (StCr16; *n* = 53), and in Valdivia during the 2016 (Vald16; *n* = 73) and 2018 (Vald18; *n* = 79) seasons. Box-and-whisker plots show the 25th percentile (lower box limit), median (black line), and 75th percentile (upper box limit). Whiskers represent the 10th and 90th percentiles, and the black line within each box marks the mean. Panels (**A**,**C**,**E**,**G**) compare locations (StCr16 vs. Vald16), whereas panels (**B**,**D**,**F**,**H**) compare seasons (Vald16 vs. Vald18). Significant differences were evaluated using Student’s *t* test; *p* values are coded as *p* < 0.01 (**), *p* < 0.001 (***), and ns = not significant.

Fruits across all populations weighed between 3.0 and 44.6 g (Figure 4A,B; Table S1), with a mean of 21.0 ± 0.6 g (CV = 43.4%; Table S1), showing a normal distribution in each population. *Lardizabala biternata* fruits ranged from 20.1 to 83.4 mm in length (FL), with an average of 54.2 ± 1.0 mm (CV = 24.1%; Figure 4C,D; Table S2). Fruit width (FW) and fruit height (FH) averaged 24.5 ± 0.2 mm (CV = 12.7%) and 23.1 ± 0.2 mm (CV = 13.0%), respectively (Figure 4E–H; Table S2). Fruit morphology differed significantly among dimensions (FL vs. FW vs. FH; ANOVA: *p* < 0.001). FL values were consistently higher than both width and height in the same fruits, whereas FW and FH showed similar magnitudes (Figure S4). Estimated traits, including fruit diameter (FD) and fruit volume (FV), averaged 23.8 ± 0.2 mm (CV = 12.5%) and 25.379 ± 707 mm^3^ (CV = 39.9%), respectively (Table S2).

#### 3.3.1. Hypothesis 1: Spatial (StCr16 vs. Vald16) Variation in Fruit Morphology (Weight and Size)

*L. biternata* fruits had no significant difference in fresh weight (IFW) between StCr16 and Vald16 [*t*_(124)_ = −0.08; *p* > 0.05] (Figure 4A), indicating similar overall mass. Regarding fruit size, no significant differences were detected in fruit length, but the other dimensions varied significantly (Figure 4C,E,G). Fruits from Santa Cruz were wider [FW: +9.2%; *t*_(124)_ = −3.64; *p* < 0.001] and higher [FH: +11.6%; *t*_(124)_ = −4.29; *p* < 0.001] than those from Valdivia (Figure 4E,G). Santa Cruz fruits also showed greater diameter (FD) and volume (FV), by 10.4% and 18.7%, respectively (Table S2).

#### 3.3.2. Hypothesis 2: Seasonal (Vald16 vs. Vald16) Variation in Fruit Morphology (Weight and Size)

Fruits from Vald18 (25.9 ± 1.0 g) were significantly heavier than those from Vald16 (17.9 ± 0.9 g) [IFW: *t*_(150)_ = −6.02; *p* < 0.001] (Figure 4B). FL increased by 13.0% in 2018 [FL: *t*_(150)_ = −3.18; *p* < 0.01] (Figure 4D), whereas FW (+12.7%) and FH (+12.1%) were also significantly greater in Vald18 than in Vald16 (*p* < 0.001 for both; Figure 4F,H). Fruits from Vald18 were significantly greater in FD (+12.2%) and FV (+39.4%) than those from Vald16 (Table S2). Overall, these findings suggest interannual variation in fruit size and weight.

### 3.4. Weight and Size Associations in L. biternata Fruits

**Figure 5:**
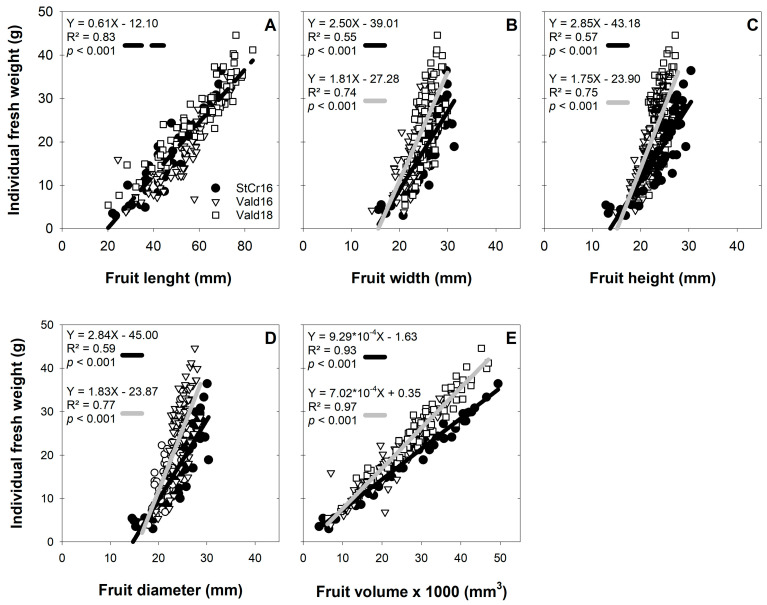
Association of morphological traits of *Lardizabala biternata* fruits. (**A**) Association of the individual fruits weight (IFW) with the length (FL), (**B**) width (FW), (**C**) height (FH), (**D**) diameter (FD) and (**E**) volume (FV) de same fruits of *L. biternata* collected close Santa Cruz city during the 2016 season (StCr16, close circle), and Valdivia city during 2016 (Vald16, open triangle) and 2018 (Vald18, open square) seasons. Lines represent the regression analysis, total data (StCr16 plus Vald16 plus Vald18, dotted line), Santa Cruz data (StCr16, grey line) and Valdivia data (Vald16 plus Vald18, grey line).

Linear regression analyses revealed significant and positive relationships between IFW and all morphological traits (Figure 5). The association between IFW and FL was captured by a single regression line (Figure 5A), whereas two distinct slopes emerged in other relationships (e.g., IFW–FW and IFW–FH; Figure 5B–E), suggesting modulation by local or seasonal factors in these morphology–weight associations. The strongest association was observed between IFW and fruit volume, highlighting volume as the best predictor of fruit biomass (Figure 5E). Relationships between IFW and both FW and FD exhibited steeper slopes than those observed in the IFW–FV relationship (Figure 5C,D).

### 3.5. Internal Fruit Composition

**Figure 6:**
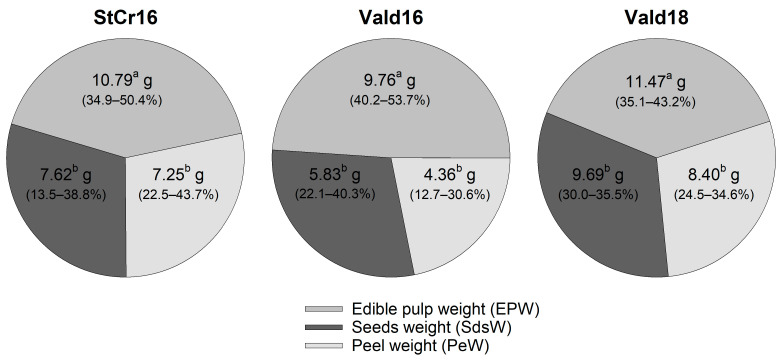
Weight of edible pulp, seeds, and peel of *Lardizabala biternata* fruits. The average weight of edible pulp (EPW), seeds (SdsW), and peel (PeW) of *L. biternata* fruits collected close Santa Cruz city during the 2016 season (StCr16), and Valdivia city during 2016 (Vald16) and 2018 (Vald18) seasons. Contributions of EPW, SdsW, and PeW to individual fresh weight in percentage. Different letters indicate significant differences between fruit components (EPW vs. SdsW vs. PeW) after ANOVA and Tukey’s test (*p* < 0.05).

Subgroups of fruits had average fresh weights of 25.7 g (range: 11.9–36.4 g) in StCr16, 20.0 g (range: 12.1–30.0 g) in Vald16, and 29.6 g (range: 18.1–39.9 g) in Vald18. All fruit consistently displayed three anatomical components: white pulp, multiple black seeds, and a thin peel (Figure 2D–F). The EPW was the main contributor to fruit weight (range: 34.9–53.7%), averaging 10.5 ± 0.5 g (CV = 30.1%; Figure 6). Seed and peel weights ranged from 1.6 to 13.6 g (range: 13.4–40.3%) and from 1.7 to 10.2 g (range: 12.7–43.7%), respectively (Table S3). The ANOVA test showed significant differences in component weights in StCr16 (*p* < 0.01), Vald16 (*p* < 0.001) and Vald18 (*p* = 0.71) (Figure 6, Table S3). The sum of EPW and PeW [(EP + Pe)W] averaged 16.7 ± 0.8 g (range: 8.2–27.0 g), with a coefficient of variation of 31.5% (Table S3).

**Figure 7:**
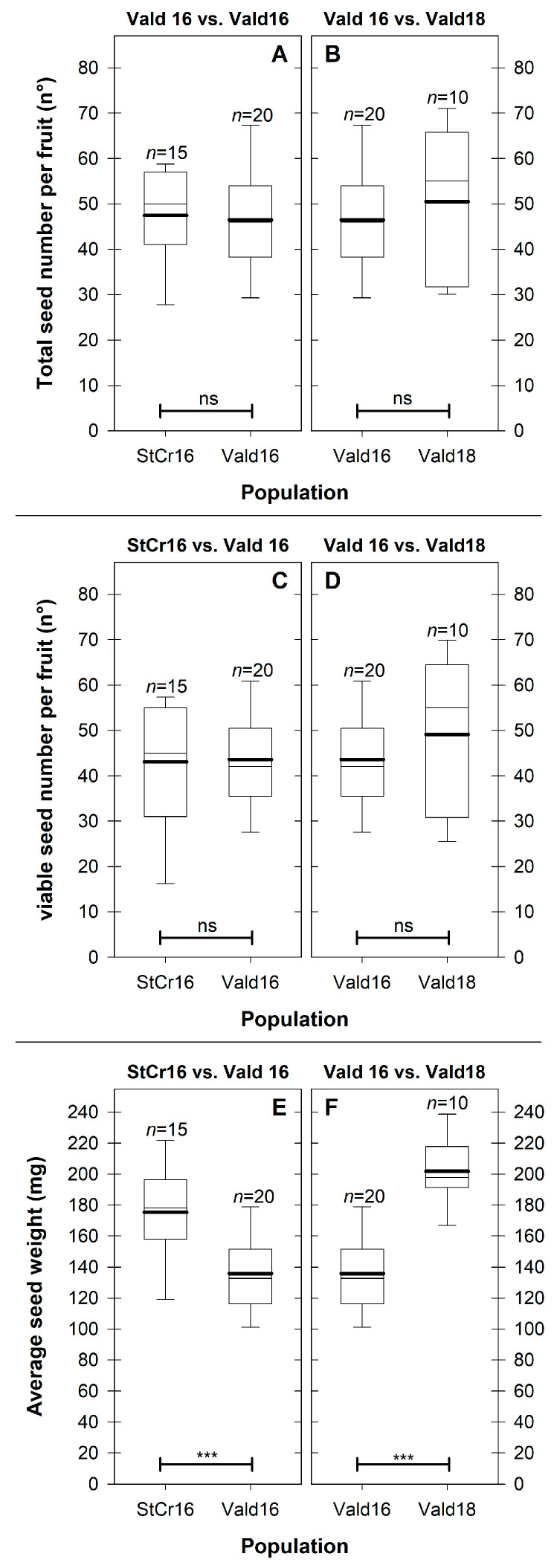
Seeds traits of *Lardizabala biternata* fruits. (**A**,**B**) Total seed number per fruit (n°), (**C**,**D**) viable seed number per fruit (n°) and (**E**,**F**) average seed weight (mg) of *L. biternata* fruits collected close Santa Cruz city during the 2016 season (StCr16; 15 fruits), and Valdivia city during 2016 (Vald16; 20 fruits) and 2018 (Vald18; 10 fruits) seasons. Box-and-whisker plots depict the parameters; the lower limit of the box indicates the 25th percentile, the black line represents the median (50th percentile), and the upper limit of the box indicates the 75th percentile. The error bars on either side of the box indicate the 10th and 90th percentiles. The black line within the box marks the mean. (**A**,**C**,**E**) show differences between locations (StCr16 vs. Vald16), while (**B**,**D**,**F**) show differences between seasons (Vald16 vs. Vald18). Significant differences were evaluated with Student’s *t*-test. Only highly significant results (*p* < 0.001) are shown and denoted by ***. ns indicates not significant.

Seeds contained in the fruit, typically arranged in six rows in transverse plane (Figure 2D,E and Figure 3D). On average, fruits contained 48 ± 2 seeds (range: 20–71 seeds; CV = 26.4%), indicating substantial variation between fruits (Figure 7A,B, Table S4). Of these, 93.1% were viable (45 ± 2 seeds; CV = 30.6%) and 6.9% were non-viable (3.0 ± 0.7 seeds; CV = 156.3%), as shown in Figure 7C,D (Table S4). Additionally, average seed dry weight (ASdW) ranged from 94.0 to 240.2 mg, with a mean of 163.7 ± 5.8 mg (CV = 23.5%; Figure 7E,F).

#### 3.5.1. Hypothesis 1: Spatial (Stcr16 vs. Vald16) Variation in Internal Fruit Components (Edible Pulp, Seed and Peel)

Significant differences in peel (+66.4%) and seed (+30.6%) weights were also observed between sampling locations (Table S5). Compared to Vald16, fruits from Santa Cruz had significantly heavier peels [PeW: *t*_(33)_ = −4.16; *p* < 0.001] and seeds [SdsW: *t*_(33)_ = −2.30; *p* < 0.05]. However, edible pulp weigh did not differ significantly between sampling locations [EPW: *t*_(33)_ = −0.97; *p* > 0.05]. The combined weight of edible pulp and peel was also significantly higher in StCr16 (+27.8%) than in Vald16 [(EP + Pe)W: *t*_(33)_ = −2.45; *p* < 0.05]. Similarly, average seed weight was significantly higher in StCr16 than in Vald16, with a 29.1% difference between locations [ASdW: *t*_(33)_ = −3.91; *p* < 0.001], suggesting environmental effects on seed development (Figure 7E). In contrast, no significant differences were detected in total seed number (TSdn°), viable seeds (VSdn°), or non-viable seeds (FSdn°) between locations (*p* > 0.05; Figure 7A,C, Table S4).

#### 3.5.2. Hypothesis 2: Seasonal (Vald16 vs. Vald18) Variation in Internal Fruit Components (Edible Pulp, Seed and Peel)

Fruits of *L. biternata* harvested in two different seasons showed significantly higher peel (+92.6%) and seed (+66.2%) weights in Vald18 compared to Vald16 [PeW: *t*_(28)_ = −4.77; *p* < 0.001; SdsW: *t*_(28)_ = −4.73; *p* < 0.001] (Table S5). Pulp weight did not differ between seasons [EPW: *t*_(28)_ = −1.44; *p* > 0.05] (Table S5). The combined weight of pulp and peel was 40.7% higher in Vald18 [*t*_(28)_ = −2.94; *p* < 0.01]. Average seed weight also increased by 48.5% in Vald18 [ASdW: *t*_(28)_ = −7.02; *p* < 0.001] (Figure 7F). In contrast, total seed number, viable seeds, and non-viable seeds showed no seasonal differences (*p* > 0.05; Figure 7B,D; Table S4).

### 3.6. Edible and Structural Components and Seed Characteristics Associations in L. biternata Fruits

**Figure 8:**
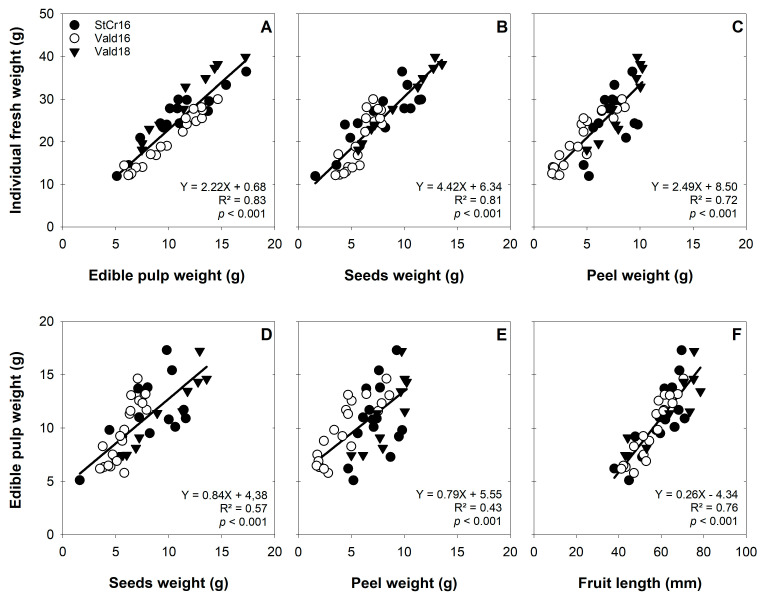
Association of internal structures of *Lardizabala biternata* fruits. Association of the individual fruits weight (IFW) with the (**A**) edible pulp weight (EPW), (**B**) seeds weight (SdsW) and (**C**) peel weight (PeW). Simultaneously, the association of the EPW with the (**D**) SdsW, (**E**) the PeW and (**F**) fruit length (FL) of *L. biternata* fruits collected close Santa Cruz city during the 2016 season (StCr16), and Valdivia city during 2016 (Vald16) and 2018 (Vald18) seasons. Solid lines show the regression analysis.

Linear regression analyses revealed significant correlations between individual fresh weight (IFW) and the weight of all fruit components across locations and seasons (Figure 8A–C). The strongest association was between IFW and EPW (R^2^ = 0.83, *p* < 0.001), followed by SdsW (R^2^ = 0.81, *p* < 0.001) and PeW (R^2^ = 0.72, *p* < 0.001) (Figure 8A–C). EPW also correlated positively with SdsW (Figure 8D), PeW (Figure 8E), and FL (Figure 8F). These patterns suggest stable internal relationships despite variation in absolute values and highlight the predictive value of morphological traits for estimating pulp yield. IFW also correlated positively with total seed number (TSdn°: Y = 0.46X + 2.12, R^2^ = 0.57, *p* < 0.001) and viable seeds (VSdn°: Y = 0.44X + 4.20, R^2^ = 0.62, *p* < 0.001). The association with average seed weight (ASdW) was weaker (Y = 8.52 × 10^−2^X + 10.0, R^2^ = 0.18, *p* < 0.05).

### 3.7. Correlations Between the Characters and Principal Components Analysis

**Figure 9:**
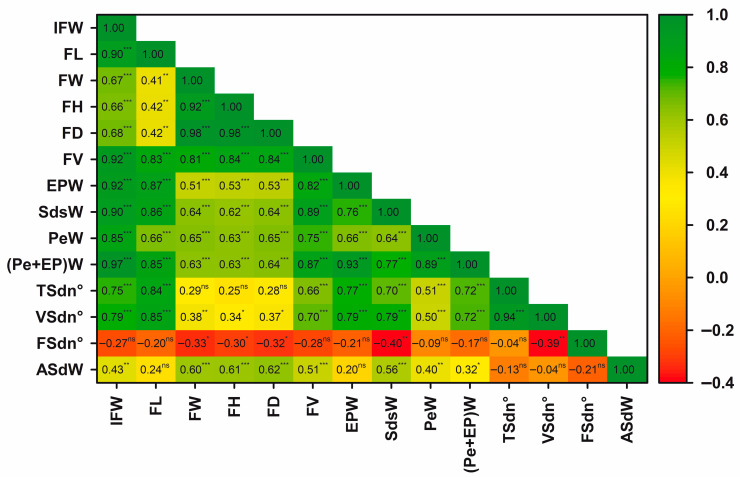
Correlation heat map showing the relationship between morphological traits of *Lardizabala biternata* fruits. The heat map displays the correlation coefficient values between pairs of traits. Data were registered for fruits of *L. biternata* collected close Santa Cruz city during 2016 season (StCr16), as well as Valdivia city during 2016 (Vald16), and 2018 (Vald18) seasons. IFW: Individual fresh weight; FL: fruit length; FW: fruit width; FH: fruit height; FD: fruit diameter; FV: fruit volume; EPW: edible pulp weight; SdsW: seeds weight; PeW: peel weight; (EP + Pe)W: edible pulp plus peel weight; TSdn°: total seed number per fruit; VSdn°: viable seed number per fruit; FSdn°: non-viable seed number per fruit; and ASdW: average seed weight. ns, not significantly different at *p* > 0.05; *, ** and *** different at *p* < 0.05, < 0.01 and < 0.001, respectively.

Correlation analyses (heatmap, Figure 9) revealed strong positive associations among key traits across all populations. IFW was significantly correlated with FL, FV, EPW, and (EP + Pe)W, but not with FSdn°. The EPW was positively correlated with FL, FV, and VSdn°, and negatively with FSdn°. Furthermore, ASdW was positively correlated with FW and FH, but negatively with TSdn° and VSdn° (Figure 9).

**Figure 10:**
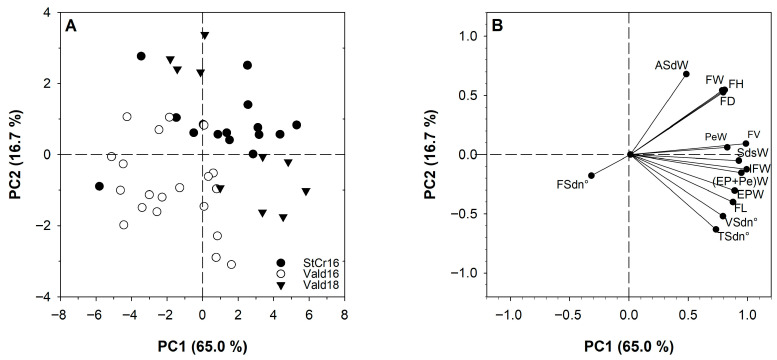
Principal component analysis of morphological traits of *Lardizabala biternata* fruits. Fruits collected close Santa Cruz city during 2016 (StCr16) and Valdivia city 2016 (Vald16) and 2018 (Vald18) in the PC1, PC2 plane: score plot (**A**) and correlation loadings (**B**). IFW: Individual fresh weight; FL: fruit length; FW: fruit width; FH: fruit height; FD: fruit diameter; FV: fruit volume; EPW: edible pulp weight; SdsW: seeds weight; PeW: peel weight; (EP + Pe)W: edible pulp plus peel weight; TSdn°: total seed number per fruit; VSdn°: viable seed number per fruit; FSdn°: non-viable seed number per fruit; and ASdW: average seed weight.

Principal component analysis (PCA) revealed distinct patterns of covariation among fruit morphological traits in *L. biternata*. The first two principal components (PC1 and PC2) accounted for 81.7% of the total phenotypic variation, with PC1 explaining 65.0% and PC2 16.7% (Figure 10A, Figure S5). In the analysis, eigenvalues greater than 1.0 were used to determine which principal components (PCs) to include (Table S6). The PCA biplot (Figure 10B) showed that PC1 was predominantly associated with traits linked to fruit mass and size, such as IFW, EPW, SdsW, PeW, FL, and FV. In contrast, PC2 captured variation associated with structural dimensions such as FW, FH, FD and ASdW (Figure 10B).

Notably, IFW clustered closely with EPW, PeW, and SdsW, indicating strong co-variation among these traits. EPW also aligned closely with FL and VSdn°, suggesting coordinated development between pulp production and fruit elongation. Conversely, ASdW was oriented away from seed number traits (VSdn° and TSdn°), reflecting a negative correlation that suggests a trade-off between seed size and number. These PCA results support the existence of coordinated yet distinct axes of morphological variation that structure fruit phenotypes across locations and seasons (Figure 10).

## 4. Discussion

This study presents the first comprehensive morphologic and morphometric dataset of *L. biternata* fruits, enhancing scientific understanding of their phenotypic variation. This information is crucial for identifying physiological traits, ecological value, genetic characteristics, and potential agricultural and commercial applications, among other aspects. Morphological traits of fruits are valuable not only for plant identification and classification, but they also serve as a bridge between traditional phenotyping and modern molecular genetic approaches. Morphological parameters reported in these publications (such as length and diameter), as well as other traits like skin colour and curvature, have been useful tools for characterising the genetic diversity of cucumbers [64]. When combined with genetic analysis, these traits provide a more comprehensive understanding of biodiversity and evolutionary relationships [31,32,44,65].

Although the number of fruits collected per site may appear modest, *L. biternata* is a rare and scarcely distributed species. Its populations are fragmented and often hidden within the understorey of native temperate forests [5,20,21], making access and identification particularly challenging. Locating mature individuals and harvesting fruits requires substantial sampling effort and specialised botanical knowledge, as the plant is often difficult to distinguish from surrounding vegetation. Thus, the samples analysed here represent a significant and representative effort given the ecological constraints of the species.

As emphasised in study [31], quantifying morphological and morphometric traits represents a fundamental initial step in the evaluation and classification of germplasm within *Lardizabalaceae* and related taxa. For example, such traits have been widely applied in the classification of crops like grapevine (*Vitis vinifera*), where fruit characteristics—such as colour, shape, and texture—serve to distinguish cultivars intended for wine production or fresh consumption [33,34,38]. These morphological parameters can be complemented by physicochemical and nutritional traits such as pH, water content, glucose, fructose, protein, ash, fat and other [32]. A comparable strategy has been employed in cereal crops, particularly maize, where kernel traits including length, width, thickness, and thousand-kernel weight are used to differentiate between landraces and commercial varieties [35,36,37,66].

In the case of *L. biternata*, there are currently no standardised morphological descriptors for plant structures, fruits, seeds, or other related traits. The fourteen morphological characters analysed in this study revealed substantial variation in fruit traits across both locations and seasons, along with consistent inter-trait associations. This work therefore provides the first baseline morphological dataset for the species, offering a valuable reference for germplasm evaluation, the identification of elite genotypes, and the development of agronomic and scientific strategies aimed at improving and conserving the species.

### 4.1. Weight of Lardizabala biternata Fruits

Fruit weight is considered one of the primary target traits in plant breeding. Notably, no previous studies have reported quantitative data on fruit weight in *L. biternata* [3,20,30]. Our findings show that fruits from two wild populations of *L. biternata* have an average fresh weight of 21.0 g, exhibiting high variability (CV = 43.4%) with maximum individual fruit weights of up to 44.6 g. On average, these Chilean wild fruits are lighter than those of related Asian species, such as *D. insignis* (31.4 g) and *A. quinata* (72.1 g) [39,41]. The difference becomes more pronounced when comparing *L. biternata* with genetically improved cultivars of *A. quinata*, whose fruits can reach up to 546 g under optimal conditions [39,41]. Despite these contrasts, the maximum fruit weight potential of *L. biternata* remains unknown, suggesting that future breeding efforts could aim to increase fruit mass by enhancing the contribution of edible pulp, seed, or peel, and by optimising the agronomic or nutritional factors that influence these components.

Each *L. biternata* fruit consists of three distinguishable structures: multiple black or dark brown seeds, white pulp, and a peel encasing both pulp and seeds. These components determine the individual fruit weight (IFW), although they contribute unequally and exhibit substantial variability (Figure 6). In this study, pulp was the main contributor to IFW (~50%), followed by seeds and peel. In terms of moisture content, the pulp retained the highest water content, whereas seeds represented the principal source of dry matter. When compared with related species, *L. biternata* exhibits a thinner and lighter peel, representing less than 24.5% of the total fruit weight, in contrast to 35.9% in *D. insignis* and 62.9% in *A. quinata* [39,41]. In contrast, edible pulp proportions were comparable among species, while *L. biternata* seeds were slightly heavier. These findings suggest that *L. biternata* may offer an advantage in terms of higher pulp yield and reduced peel mass, making it a promising candidate for breeding programmes aimed at improving fruit quality.

### 4.2. Edible Proportion and Nutritional Potential

To evaluate *L. biternata* fruits as a potential food source, it is essential to understand both their edible proportion and nutritional value. In this study, the ratio of edible to inedible components in *L. biternata* fruits exceeded 60%, a figure substantially higher than that reported for related species such as *A. quinata* (17–40%) and *D. insignis* (48.5%) [39,41,67]. While the pulp and peel of these Asian species are edible [35], the peel of *A. quinata* is typically avoided due to its bitterness [35]. Instead, it is mainly used in pharmaceutical applications and is generally consumed only after proper harvesting and culinary preparation [35]. By contrast, the peel of *L. biternata* is palatable and lacks the bitterness reported for *A. quinata* (personal observation). No adverse effects associated with its consumption have been documented, although no formal studies have evaluated its chemical composition or potential toxicological properties to date [18,19]. Ongoing analyses are currently being conducted to address this knowledge gap. Analysing the nutritional composition of both the peel and the edible pulp of *L. biternata* will be essential to confirm its safety and further substantiate its potential as a food source. Furthermore, identifying its bioactive compounds would add significant value, establishing the species as a novel edible and functional resource as in other species of *Lardizabalaceae* [40]. For example, *Akebia* species contain considerable amounts of alkaloids, amino acids, flavonoids, lignans, steroids, and other bioactive compounds [40]. Therefore, *L. biternata* is likely to share some of these bioactive compounds, although this remains to be investigated.

Fruit peels often contain higher concentrations of fibre, antioxidants, and phenolic compounds than the pulp, making them valuable targets for nutritional research. In the case of *L. biternata*, however, no nutritional or chemical profiles have yet been published, and the species remains largely overlooked in both nutritional and ecological contexts. We have recently initiated analyses of the pulp and seeds, but these results are still preliminary and require validation before publication. A comprehensive characterisation of the fruit’s nutritional and functional properties, including sensory, biochemical, and ethnobotanical dimensions, thus remains a key priority for future research.

### 4.3. Seeds as a Resource

Beyond the edible portion, non-edible components such as seeds may also hold significant value. Huang et al. [58] emphasised the importance of examining seed traits in *A. quinata*, a perspective that may also be relevant for *L. biternata*. Although this study primarily addressed the fruit’s food potential, future research could investigate the seeds as sources of dry matter, fatty acids, or bioactive compounds with potential pharmacological applications [68,69,70]. Supporting this line of inquiry, our work provides the first morphological characterisation of *L. biternata* seeds, including total seed weight, seed number, average seed weight, and their relationships with edible traits (Figure 7, Figure 8 and Figure 9). Understanding these associations may inform agricultural selection and breeding strategies. For example, fruit length or fresh weight at harvest could serve as predictors of seed number and weight, while seed number may indicate fertility and provide insights into pollination success, ovule development, and reproductive performance.

This study contributes to the current understanding of fruit weight in *L. biternata*. However, the available information remains limited. Further research is needed to deepen our knowledge of the biochemical and nutritional properties of the pulp, peel, and seeds. In addition, it is crucial to identify genotypes that exhibit an optimal balance among these fruit components or to selectively enhance specific traits through breeding efforts.

### 4.4. Fruit Size of Lardizabala biternata

To date, fruit length is the only morphological trait of *L. biternata* fruits documented in scientific literature. These measurements are, however, based on an unknown number of specimens and so lack reliability and statistical robustness. A botanical guide reported a length of approximately 50 mm [3], while a culinary (non-scientific) guide described fruit lengths of up to 80 mm [25]. Our study refines this characterisation by revealing the underlying complexity of this trait. Specifically, we found an average fruit length of 54.2 mm, exceeding previously reported values, with over 52% of fruits surpassing this average and some reaching more than 80 mm (Figure 4C,D). Notably, the longest *L. biternata* fruits observed in this research approached the length of Asian species; but the average fruit length remained well below that of *A. quinata* (<104 mm) and *D. insignis* (<111 mm) [39,41].

This study also introduces two morphological traits, i.e., fruit width and height, which have not been previously documented for the species. These measurements provide mathematical estimates of derived traits such as fruit diameter and volume, which are valuable for future research. Fruit length showed the greatest variability (CV = 24.1%), whereas width (24.5 mm) and height (23.1 mm) showed lower absolute values and reduced variability (CV < 13.0%) (Figure 4E–H, Table S2). Both width and height were responsive to environmental factors, with higher values recorded in fruits collected during the StCr16 and Vald18 seasons (Figure 4E–H). The minimal difference between width and height suggests that these measurements could be integrated into a single descriptor (e.g., diameter), or used to estimate fruit volume, approaches that may facilitate morphological comparisons in future studies [71,72,73,74,75].

Fruits of *L. biternata* are best described as oblong to sub-oblong in shape (Figure 2A–C). Fruit size and shape are determined by genetic mechanisms and modulated by environmental conditions such as temperature, light, and humidity [76,77,78]. In many species, elongation is represented by fruit length, while radial growth is reflected in fruit diameter [76]. In *L. biternata*, two distinct axes of growth can be identified: longitudinal elongation and transverse expansion. Longitudinal growth correlates with fruit weight and seed number; specifically, a greater number of viable seeds along the longitudinal axis is associated with longer fruits. In contrast, we observed a consistent radial arrangement of six seeds extending from the central axis toward the pericarp (Figure 2E and Figure 3D). This configuration suggests that average seed size and weight may constrain transverse growth, limiting variation in width, height, and diameter.

### 4.5. Impact of Climatic Conditions

At present, *L. biternata* lacks formal physiological or phenological studies documenting the timing and synchrony of its reproductive stages, including flowering, fruit set, and ripening [3,4]. Preliminary field observations suggest flowering may occur between April and May, but these reports remain anecdotal and require systematic validation. Our decision to use a March-to-February climatic window assumed of annual periodicity, aiming to capture the environmental conditions influencing fruit development from one harvest cycle to the next. This is consistent with phenological models for other temperate lianas and perennial climbers [42,79]. Consequently, while our analyses offer novel insights into climate–fruit trait relationships, these associations should be interpreted considering the current knowledge gaps and the need for future studies to empirically define the phenophases of *L. biternata* across its range.

Given the broad latitudinal range of *L. biternata*, its populations are exposed to diverse climatic conditions, which may influence morphological traits [53,56,80]. Our results showed significant differences in precipitation and temperature between the two study sites (Santa Cruz vs. Valdivia), with less pronounced seasonal climatic variability in Valdivia (Table 1). Santa Cruz generally experiences lower rainfall and higher temperatures; however, during the study period, an unusual increase in spring precipitation was recorded (Figure S1). These climatic conditions likely affect soil moisture, water flow, and other environmental variables, which in turn can influence key physiological processes such as seed germination and plant development. Traits like fruit weight and length may be especially susceptible during sensitive phenological stages such as flowering and pollination. In extreme scenarios, such as temperatures exceeding 35 °C, pollen viability and pollen tube growth may be compromised, as documented in species like tomato [79,81].

In Central Chile, low rainfall combined with high temperatures, can reduce water availability and increase evapotranspiration, negatively affecting fruit development [82]. Nevertheless, *L. biternata* is predominantly found in evergreen to semi-deciduous forests, ravines, and moist understory environments [4,14,26]. These habitats may function as microrefugia, climatically buffered sites that provide stable environmental conditions and promote species persistence under otherwise unfavourable macroclimatic change [83,84,85]. Within these microrefugia, traits such as individual fruit weight and length may be more strongly shaped by short-term biological factors, such as the success of pollination, rather than by large-scale climatic variability. Conversely, more conservative traits, such as fruit width, height, and average seed weight, exhibited lower variability and may reflect radial growth processes that are more sensitive to cumulative effects of increased temperature and reduced humidity.

The limited number of populations assessed in this study restricts our capacity to evaluate the genetic diversity and phenotypic plasticity of *L. biternata*, especially in traits associated with drought tolerance, thermal stress, pollination efficiency, and flowering duration [86]. The role of Chile’s diverse ecoregions, shaped by complex climatic and altitudinal gradients, also remains largely unexplored and deserves further investigation.

From an ecological perspective, the smaller fruit size of *L. biternata* compared to another *Lardizabalaceae*, i.e., *A quinata*, may reflect adaptation to local dispersers and habitat constraints. In southern Chile, *L. biternata* is arguably dispersed by the South American grey fox (*Lycalopex griseus*), which favours moderate-sized fleshy fruits and contributes to seed dispersal via endozoochory [30]. Unlike *A. quinata*, which thrives in cultivated or disturbed environments, *L. biternata* grows in dense temperate rainforests as a woody vine, often under low-light conditions. Such habitats may impose constraints on fruit size and investment, favouring smaller, resource-efficient fruits that are still attractive to vertebrate dispersers. These ecological pressures could drive the fruit morphological differences observed between the two species.

### 4.6. Evaluation of Hypotheses

This study was guided by two main hypotheses: (1) that fruit morphological traits vary significantly between two geographically distant populations, i.e., Santa Cruz (warmer and drier) and Valdivia (cooler and wetter), due to climatic differences; and (2) that morphological traits also differ between seasons within the Valdivia population, reflecting interannual environmental variation.

The results strongly support both hypotheses. Regarding the spatial component, fruits from Valdivia and Santa Cruz exhibited significant differences in width, height, diameter, volume, and seed weight, with Valdivia fruits showing smaller transverse dimensions and lighter seeds in 2016 (Figure 4 and Figure 6). These differences are consistent with local climatic regimes, suggesting an effect of environmental conditions on fruit development. As for the temporal component, fruits collected in Valdivia in 2018 were significantly larger and heavier than those from 2016. This finding points to interannual climatic variation, particularly changes in temperature and precipitation, as a driver of phenotypic plasticity.

Together, these findings confirm that both spatial and seasonal variation influence fruit morphology in *L. biternata*, reflecting the species’ potential sensitivity to environmental factors and highlighting its phenotypic plasticity. The significant variation observed both between populations (Santa Cruz vs. Valdivia) and between seasons within the same population (Vald16 vs. Vald18) underscores the existence of a plastic phenotypic response to environmental conditions in *L. biternata*. Such intra- and inter-population variation, coupled with strong trait correlations, supports the notion that fruit morphology in this species is modulated by both genetic and environmental factors. This variation constitutes not only evidence of adaptive plasticity but also a valuable reservoir of phenotypic diversity for future selection. As demonstrated in other perennial crops [31,35,38,43], the ability to detect and quantify this variation at early stages of domestication enables the identification of genotypes with favourable trait combinations, enhancing the efficiency of breeding programmes. The study of morphological traits in fruits and plants of *Punica granatum* makes it possible to distinguish contrasting genotypes in terms of yield, growth, canopy coverage, and other agronomically valuable parameters [65]. In the context of *L. biternata*, traits such as fruit length, pulp content, and seed number may be targeted to balance fruit quality and reproductive success under variable climatic scenarios.

### 4.7. Correlations Between Phenotypic Traits

The correlation matrix (Figure 9) reveals a set of positive and statistically significant associations among key morphological traits in *L. biternata*. Fresh fruit weight exhibited strong positive correlations with pulp content, fruit length, width, and height. These patterns suggest a coordinated development of fruit dimensions and pulp accumulation, indicating that selecting for increased fruit size may simultaneously enhance pulp yield, an essential trait for both fresh consumption and potential industrial applications. The consistent positive correlations among fruit dimensions (length, width, and height) also suggest an allometric growth pattern, where increases in one axis are proportionally reflected in the others [42,87]. Such interdependence supports the use of indirect selection strategies, where improving a more heritable or easily measured trait (e.g., fruit length) can indirectly enhance more complex traits (e.g., pulp yield) [31,43,44].

Averaged seed weight displayed weak correlations with fresh fruit weight and was not significantly related to pulp content (Figure 9). These findings suggest that seed number along the longitudinal axis may be the main driver of increased fruit weight and pulp content, whereas seed size and radial arrangement may constrain transverse growth (e.g., width and diameter). This spatial differentiation reflects the internal organisation of *L. biternata* fruits, where radially arranged seeds appear to limit peel and pulp expansion. Optimising the balance among seed number, size, and spatial configuration could therefore support the production of larger fruits with fewer or lighter seeds, traits desirable for both breeding and consumer preference [31,43,44].

The inverse association observed between seed weight and seed number suggests that plants such as *L. biternata* may prioritise either rapid growth or propagation through the production of many small seeds. Commonly, a fruit, organism, or ecosystem cannot maximize all desirable traits or functions simultaneously due to limited resources and trade-offs [88]. In this case, producing a high number of seeds, limiting the seed weight, combined with an effective dispersal vector may enhance reproductive success [30,89], specially under the increasingly variable climatic conditions of Chile. From an agricultural perspective, considering seed traits as breeding targets will require identifying the variables that determine the optimal relationship between seed weight and seed number [90], either to improve reproductive efficiency or to evaluate seeds as a potential source of resources.

As result, the correlation matrix suggests potential genetic linkages among associated traits [91]. Overall, the correlation structure provides a strong foundation for the rational selection of genotypes with superior fruit attributes, emphasising the value of morphological characterisation in early-stage domestication and breeding efforts.

### 4.8. Correlations Between Phenotypic Traits Morphological Traits as Physiological and Agricultural Indicators in Lardizabala biternata

Currently, the Edinburgh Botanic Garden maintains a section dedicated to Chilean plants, which includes *L. biternata*. This demonstrates the feasibility of ex situ cultivation, even beyond Chilean territory. The development and implementation of controlled cultivation strategies for this species would provide an ideal platform for future research initiatives. Achieving this will require the integration of botanical knowledge, research commitments, and supportive development policies. A first step would be defining the physiological requirements for germination and propagation, as well as the environmental conditions (soil, light, water, among others) that favour plant growth and development. Also, adequate infrastructure and institutional support would be critical to enable the establishment and long-term funding of breeding and improvement programs. Considering the challenges associated with accessing wild populations, developing ex situ collections under controlled environmental conditions emerge as a strategic priority. These collections would enable the monitoring of reproductive cycles, facilitate controlled experiments on fruit and seed development, and support propagation efforts. Establishing such collections will require coordinated institutional efforts, long-term funding, and infrastructure adapted to the species’ physiological requirements. Nonetheless, the potential benefits for research, conservation, and sustainable use, strongly justify the investment. Improving the understanding of this plant and assessing progress in its genetic enhancement, will require identifying physiological parameters that reflect developmental variation, such as the morphological traits described in this study.

In addition to morphological characterisation, advancing the introduction of *L. biternata* into cultivation systems will require detailed studies on its reproductive biology. This includes understanding the timing and synchrony of flowering in male and female individuals, pollination mechanisms, fruit set dynamics, and seed viability under different environmental conditions. Such information is fundamental for selecting high-performing genotypes, optimising agronomic management, and enhancing productivity under cultivation. These reproductive traits are known to be sensitive to environmental factors such as temperature, photoperiod, and water availability [42,79], which may strongly influence developmental events including flowering, fertilisation, and fruit development.

Phylogenetic studies comparing *L. biternata* with related South American and Asian genera (e.g., *Akebia*, *Stauntonia*, *Boquila*, *Decaisnea*) may clarify evolutionary patterns within *Lardizabalaceae* and offer valuable insights for both conservation and domestication strategies. Previous work by Kofuji et al., (1994) [29] has shown the phylogenetic proximity between South American and East Asian genera, providing a framework for exploring convergent or divergent fruit traits. Furthermore, population-level genetic characterisation in Chile would help assess diversity, identify elite genotypes, and support the development of breeding programmes tailored to different environments.

Multiple studies have shown that morphological and physiological traits are closely interconnected, reflecting internal plant processes and responses to environmental cues. In *L. biternata*, the consistent expression and measurable variation in traits such as fruit size, weight, and edible proportion underscore their relevance not only for understanding plant development and stress response but also as practical indicators for selection in early-stage breeding programmes. As demonstrated in crops like wheat, morphological traits can be used as indirect indicators of physiological functions such as source–sink dynamics, carbon allocation, and reproductive efficiency [42].

In *L. biternata*, fruit length and diameter correlate with pulp content and seed traits, indicating that they are influenced by underlying physiological processes such as carbohydrate translocation and water regulation [45,92,93,94]. Fruit size and shape, being readily observable, can serve as proxies for complex physiological phenomena [31,43,44,45,92]. These traits thus play a dual role: as indicators of physiological performance and as selection criteria for genotypes with high yield and quality. Their responsiveness to environmental stressors such as drought and temperature fluctuations also makes them valuable for evaluating genotype adaptability under climate change [81,95,96,97]. For example, plant morphological traits can serve as sensitive bioindicators of thermal stress and increasing temperatures [98,99]

From an agricultural standpoint, morphological traits have immediate applications. Larger fruit size, a higher pulp-to-seed ratio, and consistent seed weight can enhance yield, simplify postharvest handling, and improve storage potential [100,101,102,103]. Traits like fruit length, which may predict seed number, and diameter, which relates to seed weight, can serve as selection targets for breeding programmes aimed at fresh consumption, propagation, or processing.

Despite the morphological richness observed in this study, *L. biternata* remains underexplored in terms of its nutritional and pharmacological properties. This contrasts with better-known Asian relatives such as *A. quinata*, which contain essential minerals, triterpenes, anti-inflammatory polysaccharides, and bioactive saponins [58,104,105]. Several of these compounds exhibit promising antibiotic, anticancer, and industrial applications, including biofuel production. The bioactive potential reported in related species, *L. biternata* represents a promising but largely untapped resource. While it is undoubtedly relevant to consider the most recent literature on *L. biternata*, the knowledge gap surrounding this species is so extensive that direct comparisons with contemporary studies offer limited insight. In this context, a retrospective examination of historical records, or even the replication of earlier studies, may in fact be more valuable for clarifying several still unresolved aspects. To date, only one study has reported the extraction of oleanolic acid from its leaves [27], limiting our understanding of its functional potential. This gap in knowledge highlights the need for integrative research that combines morphological characterisation with nutritional, metabolomic, and pharmacological profiling of its fruit tissues.

## 5. Conclusions

This study reports fourteen morphological traits of *L. biternata* fruits, aligned with descriptors commonly used for other species in the *Lardizabalaceae* family. These include morphometric, gravimetric, and numeric variables, which exhibited varying levels of phenotypic variation. On average, fruits weighed 21.0 g, measured 54.2 mm in length and 23.8 mm in diameter. The edible pulp averaged 10.5 g, accounting for 44.4% of total fruit weight.

Statistical analyses revealed significant variation among fruit traits and between distinct trait categories (e.g., fruit length vs. width vs. height; pulp vs. seed vs. peel weight). Correlation analyses highlighted both positive and negative relationships among the traits. Fruit weight was positively associated with key attributes such as pulp weight, fruit length, and seed number and weight. In contrast, fruit diameter (width and height) was more strongly correlated with average seed weight.

Following previous studies on related fruits such as *A. quinata*, one of the primary objectives of breeding is to identify or select fruits with greater growth and weight potential, as well as higher edible pulp content. At the same time, it is essential to select genotypes with resistance to water stress, given the projected future water shortages. However, before defining breeding strategies for *L. biternata*, the nutritional properties and bioactive compounds of its edible pulp, peel, and seeds should first be characterised.

These findings provide foundational knowledge on *L. biternata* fruit morphology, encompassing trait variability and inter-trait relationships. They offer valuable phenotypic parameters to support the identification of elite genotypes, guide breeding and conservation strategies, and inform physiological and morphological assessments. In the absence of prior data on fruit traits, this work advances efforts to evaluate the species’ agricultural potential and promote its sustainable use.

## Figures and Tables

**Table 1:** Maximum (T°max), minimum (T°min), and mean (T°mean) temperatures (°C), and accumulated rainfall (mm) recorded in Santa Cruz and Valdivia during the fruit growth period of *Lardizabala biternata*.

Season	Location	Season Time	Data Harvest	T°_max_ (°C)	T°_min_ (°C)	T°_mean_ (°C)	Rainfall (mm)
StCr16	Santa Cruz	2015/16	February	23.1	8.8	16.0	586
Vald16	Valdivia	2015/16	March	17.5	7.0	12.2	1869
Vald18	Valdivia	2017/18	March	16.6	6.9	11.7	2119

## Data Availability

All data supporting the findings of this study are available within the article and its Supplementary Materials.

## Supplementary Materials

The following supporting information can be downloaded at: https://www.mdpi.com/article/10.3390/plants14203126/s1, Figure S1: Temperatures and rainfalls. Minimum (close triangle plus black dotted line), maximum (close circle plus black line), and mean temperature (open circle plus dotted line), and accumulated rainfalls (grey bars) from March to February in close Santa Cruz city during 2015–16 (A, StCr16) season, and in Valdivia city during 2015–16 (B, Vald16) and 2017–18 (C, Vald18) seasons. Figure S2: Seeds of *Lardizabala biternata* fruits. (A) Viable and (B) non-viable seeds. Figure S3: *Lardizabala biternata* fruits. The figure shows twenty *L. biternata* fruits of varying sizes. The calliper indicates a length of 5.0 cm. Figure S4: Fruit size of *Lardizabala biternata* fruits. Length (mm), width (mm) and height (mm) of *L. biternata* fruits collected close Santa Cruz city (A) during the 2016 season (StCr16), and Valdivia city during 2016 (B, Vald16) and 2018 (C, Vald18) seasons. Box-and-whisker plots depict the parameters; the lower limit of the box indicates the 25th percentile, the black line represents the median (50th percentile), and the upper limit of the box indicates the 75th percentile. The error bars on either side of the box indicate the 10th and 90th percentiles. The black line within the box marks the mean, and different letters indicate significant differences between populations after one-way ANOVA (*p* < 0.05) and Tukey’s test. Figure S5: Variance percentage accounted for three principal components in PCA for the morphological traits in *Lardizabala biternata* fruits. Table S1: Individual fresh weight (IFW) of *Lardizabala biternata* fruits. Table S2: Length (mm), width (mm), height (mm), diameter (mm) and volume (mm^3^) of *Lardizabala biternata* fruits. Table S3: Individual fresh weight (g) and weight of internal structures (g) of *Lardizabala biternata* fruits. Individual fresh weight (IFW), edible pulp weight (EPW), seeds weight (SdW), peel weight (PeW) and edible pulp plus peel weight [(EP + Pe)W]. Table S4: Total seed number (TSdn°, n°), viable seeds number (VSdn°, n°), non-viable seeds number (FSdn°, n°) and individual seed weight (ASdW, mg) of *Lardizabala biternata* fruits. Table S5: Weight of internal structures (g) of *Lardizabala biternata fruits*. Edible pulp weight (EPW), seeds weight (SdW), peel weight (PeW) and edible pulp plus peel weight [(EP + Pe)W]. Table S6: Eigenvectors of principal component axes from PCA for the morphological traits in *Lardizabala biternata* fruits.

## Abbreviations

The following abbreviations are used in this manuscript:

StCr	Santa Cruz city
Vald	Valdivia city
StCr16	Santa Cruz sampling, season 2016
Vald16	Valdivia sampling, season 2016
Vald18	Valdivia sampling, season 2018
IFW	Individual fresh weight
FL	Fruit length
FW	Fruit width
FH	Fruit height
FD	Fruit diameter
FV	Fruit volume
EPW	Edible pulp weight
PeW	Peel weight
(EP + Pe)W	edible pulp plus peel weight
SdsW	Total seed weight
TSdn°	Total seed number
VSdn°	Viable seed number
VSdn°	Nom-viable seed number
ASdW	Average seed weight
