# Supplementary material for: First Phenotypic Characterization of the Edible Fruits of Lardizabala biternata: A Baseline for Conservation and Domestication of a Neglected and Endemic Vine
Source: Plants (Basel). 2025 Oct 10;14(20):3126. doi: 10.3390/plants14203126 (PMC12567215; doi:10.3390/plants14203126)
Supplement: Supplementary file 1 [file plants-14-03126-s001.zip › plants-3817970-supplementary/Figure S1.pdf]

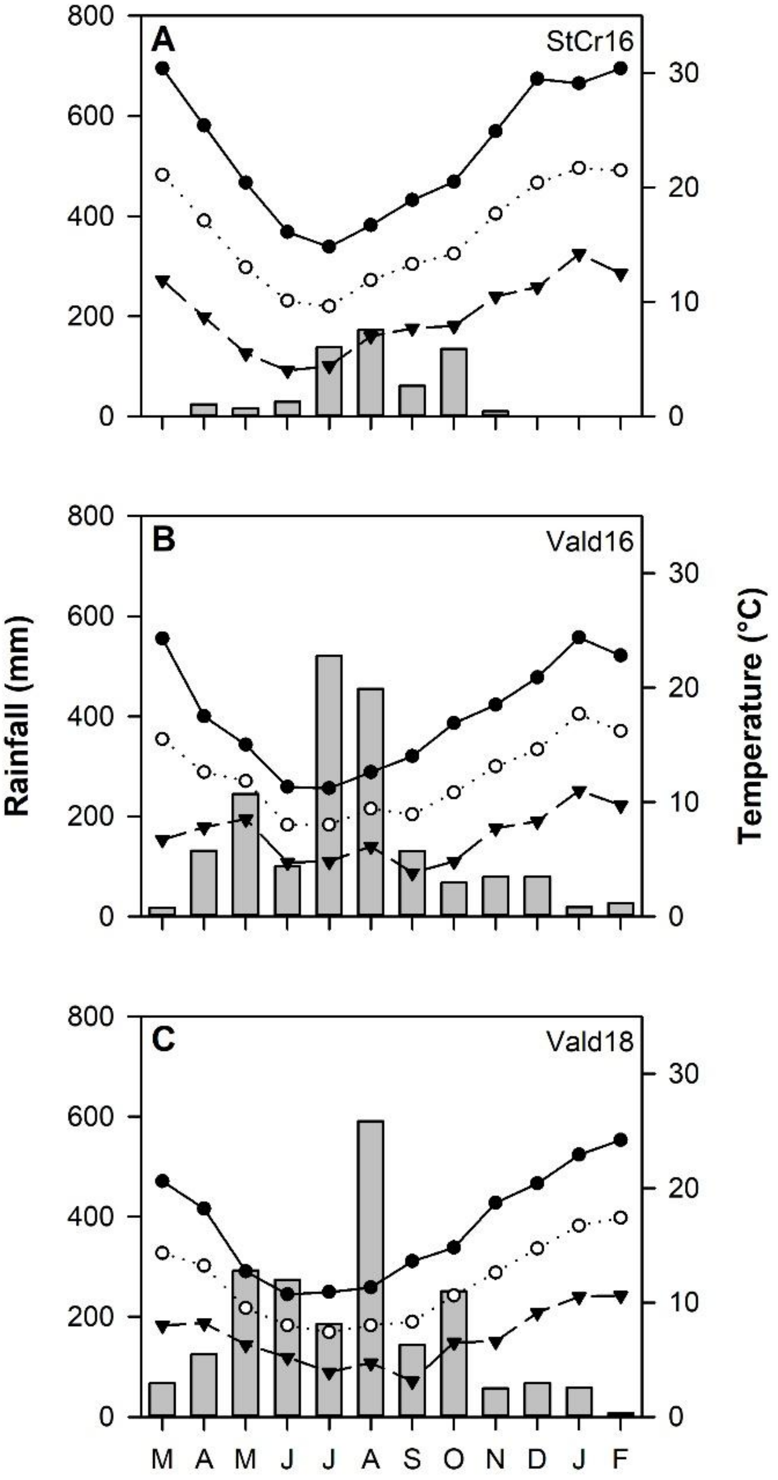

Figure S1. Temperatures and rainfalls. Minimum (close triangle plus black dotted line), maximum (close circle plus black line), and mean temperature (open circle plus dotted line), and accumulated rainfalls (grey bars) from March to February in close Santa Cruz city during 2015-16 (A, StCr16) season, and in Valdivia city during 2015-16 (B, Vald16) and 2017-18 (C, Vald18) seasons.
