# Supplementary material for: First Phenotypic Characterization of the Edible Fruits of Lardizabala biternata: A Baseline for Conservation and Domestication of a Neglected and Endemic Vine
Source: Plants (Basel). 2025 Oct 10;14(20):3126. doi: 10.3390/plants14203126 (PMC12567215; doi:10.3390/plants14203126)
Supplement: Supplementary file 1 [file plants-14-03126-s001.zip › plants-3817970-supplementary/Figure S4.pdf]

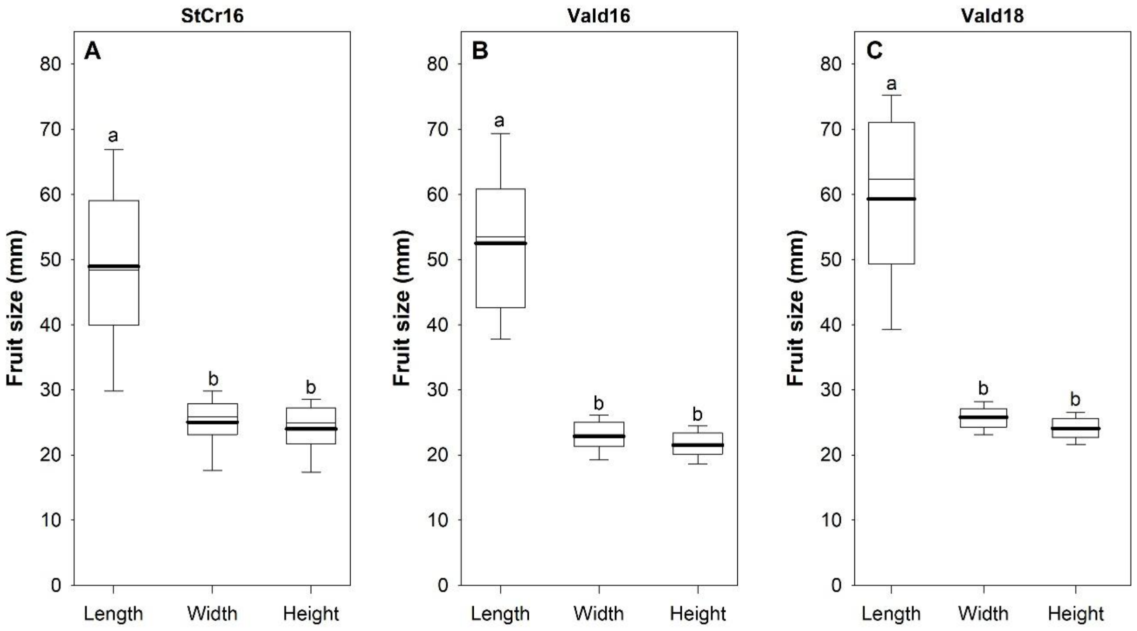

Figure S4. Fruit size of *Lardizabala biternata* fruits. Length (mm), width (mm) and height (mm) of *L. biternata* fruits collected close Santa Cruz city (A) during the 2016 season (StCr16), and Valdivia city during 2016 (B, Vald16) and 2018 (C, Vald18) seasons. Box-and-whisker plots depict the parameters; the lower limit of the box indicates the 25th percentile, the black line represents the median (50th percentile), and the upper limit of the box indicates the 75th percentile. The error bars on either side of the box indicate the 10th and 90th percentiles. The black line within the box marks the mean, and different letters indicate significant differences between populations after one-way ANOVA ($P < 0.05$) and Tukey's test.
