# Supplementary material for: First Phenotypic Characterization of the Edible Fruits of Lardizabala biternata: A Baseline for Conservation and Domestication of a Neglected and Endemic Vine
Source: Plants (Basel). 2025 Oct 10;14(20):3126. doi: 10.3390/plants14203126 (PMC12567215; doi:10.3390/plants14203126)
Supplement: Supplementary file 1 [file plants-14-03126-s001.zip › plants-3817970-supplementary/Table S1.pdf]

Table S1. Individual fresh weight (IFW) of *Lardizabala biternata* fruits.

Population	Individual fresh weight of fruits (g)					
	Fruits (n°)	Mean (g)	Min (g)	Max (g)	SD (g)	CV (%)
**StCr15**	53	18.0	3.0	36.4	8.6	47.6
**Vald16**	73	17.9	3.9	35.0	7.9	44.1
**Vald18**	79	25.9	5.4	44.6	8.5	32.7
**Total**	205	21.0	3.0	44.6	9.1	43.4
**Differences by locations (StCr16 vs Vald16)**		ns				
**Differences by seasons (Vald16 vs Vald16)**		***				

Fruits were collected from two locations: Santa Cruz and Valdivia. Data correspond to fruits harvested near Santa Cruz in the 2016 season (StCr16), and in Valdivia during the 2016 (Vald16) and 2018 (Vald18) seasons. The table shows the number of fruits (n°), individual fresh weight average (Mean), minimum (Min) and maximum (Max) weights, standard deviation (SD), and coefficient of variation (CV). Significant differences between locations (StCr16 vs Vald16) and between seasons (Vald16 vs Vald 18) were evaluated using Student's t-test ($P < 0.05$, $P < 0.01$, and $P < 0.001$) and are denoted by *, **, and ***, respectively. "ns" indicates not significant.
