# Supplementary material for: First Phenotypic Characterization of the Edible Fruits of Lardizabala biternata: A Baseline for Conservation and Domestication of a Neglected and Endemic Vine
Source: Plants (Basel). 2025 Oct 10;14(20):3126. doi: 10.3390/plants14203126 (PMC12567215; doi:10.3390/plants14203126)
Supplement: Supplementary file 1 [file plants-14-03126-s001.zip › plants-3817970-supplementary/Table S3.pdf]

Table S3. Individual fresh weight (g) and weight of internal structures (g) of *Lardizabala biternata* fruits. Individual fresh weight (IFW), edible pulp weight (EPW), seeds weight (SdW), peel weight (PeW) and edible pulp plus peel weight [(EP+Pe)W].

			Weight of internal structures (g)				
**Population**	**Morphological traits**	**Fruits (n°)**	**Mean**	**Min**	**Max**	**SD**	**CV (%)**
**StCr16**	**Individual fresh weight (g) (IFW)**	15	25.7	11.9	36.4	6.5	25.2
	**Edible pulp weight (g) (EPW)**		10.8[a]	5.1	17.3	3.3	30.7
	**Seed weight (g) (SdW)**		7.6[b]	1.6	11.6	3.1	40.3
	**Peel weight (g) (PeW)**		7.3[b]	4.7	9.8	1.6	21.5
	**Edible pulp plus peel weight (g) [(EP+Pe)W]**		18.0	10.3	26.6	4.2	23.1
**Vald16**	**Fresh weight (g)**	20	20.0	12.1	30.0	6.2	31.3
	**Edible pulp weight (g) (EPW)**		9.8[a]	5.8	14.6	2.9	29.8
	**Seed weight (g) (SdW)**		5.8[b]	3.5	7.9	1.4	24.4
	**Peel weight (g) (PeW)**		4.4[b]	1.7	8.6	2.3	53.4
	**Edible pulp plus peel weight (g) [(EP+Pe)W]**		14.1	8.2	22.9	5.1	35.8
**Vald18**	**Fresh weight (g)**	10	29.6	18.1	39.9	8.1	27.4
	**Edible pulp weight (g) (EPW)**		11.5[a]	7.5	17.2	3.4	29.6
	**Seed weight (g) (SdW)**		9.7[b]	5.7	13.6	3.1	31.9
	**Peel weight (g) (PeW)**		8.4[b]	5.0	10.2	1.9	22.0
	**Edible pulp plus peel weight (g) [(EP+Pe)W]**		19.9	12.5	27.0	5.0	25.4

Fruits were collected from two locations: Santa Cruz and Valdivia. Data correspond to fruits harvested near Santa Cruz in the 2016 season (StCr16), and in Valdivia during the 2016 (Vald16) and 2018 (Vald18) seasons. The table shows the number of fruits (n°), average of each morphological trait (Mean), minimum (Min) and maximum (Max) weights, standard deviation (SD), and coefficient of variation (CV). ANOVA test was used by differences between fruits components (EPW vs SdsW vs PeW) in each location and season. Different letters indicate significant differences between fruit components after Tukey's test ($P < 0.05$).
