# Supplementary material for: First Phenotypic Characterization of the Edible Fruits of Lardizabala biternata: A Baseline for Conservation and Domestication of a Neglected and Endemic Vine
Source: Plants (Basel). 2025 Oct 10;14(20):3126. doi: 10.3390/plants14203126 (PMC12567215; doi:10.3390/plants14203126)
Supplement: Supplementary file 1 [file plants-14-03126-s001.zip › plants-3817970-supplementary/Table S4.pdf]

Table S4. Total seed number (TSdn°, n°), viable seeds number (VSdn°, n°), non-viable seeds number (FSdn°, n°) and individual seed weight (ASdW, mg) of *Lardizabala biternata* fruits.

Morphological traits	Population	Traits of seeds				
		Mean	Min	Max	SD	CV (%)
**Total seeds (n°) (TSdn°)**	**StCr16**	47.5	11.2	23.6	20.0	60.0
	**Vald16**	46.5	11.3	24.4	28.0	68.0
	**Vald18**	50.5	17.2	34.1	30.0	71.0
	**Differences by location (StCr16 vs Vald16)**	ns				
	**Differences by seasons (Vald16 vs Vald18)**	ns				
**Viable seeds (n°) (VSdn°)**	**StCr16**	43.1	14.6	34.0	12.0	58.0
	**Vald16**	43.6	10.6	24.3	25.0	63.0
	**Vald18**	49.1	17.7	36.0	25.0	70.0
	**Differences by location (StCr16 vs Vald16)**	ns				
	**Differences by seasons (Vald16 vs Vald18)**	ns				
**Non-Viable seeds (n°) (FSdn°)**	**StCr16**	4.4	7.3	166.1	0.0	27.0
	**Vald16**	2.9	2.8	99.4	0.0	11.0
	**Vald18**	1.4	2.1	151.3	0.0	7.0
	**Differences by location (StCr16 vs Vald16)**	ns				
	**Differences by seasons (Vald16 vs Vald18)**	ns				
**Individual seed weight (mg) (ASdW)**	**StCr16**	175.4	34.4	19.6	98.3	232.6
	**Vald16**	135.9	25.6	18.8	94.0	194.6
	**Vald18**	201.8	21.2	10.5	164.7	240.2
	**Differences by location (StCr16 vs Vald16)**	***				
	**Differences by seasons (Vald16 vs Vald18)**	***				

Fruits were collected from two locations: Santa Cruz and Valdivia. Data correspond to fruits harvested near Santa Cruz in the 2016 season (StCr16), and in Valdivia during the 2016 (Vald16) and 2018 (Vald18) seasons. The table shows the average of each morphological trait (Mean), minimum (Min) and maximum (Max) weights, standard deviation (SD), and coefficient of variation (CV). Significant differences between locations (StCr16 vs Vald16) and between seasons (Vald16 vs Vald 18) were evaluated using Student's t-test ($P < 0.05$, $P < 0.01$, and $P < 0.001$) and are denoted by *, **, and ***, respectively. "ns" indicates not significant.
