# Supplementary material for: First Phenotypic Characterization of the Edible Fruits of Lardizabala biternata: A Baseline for Conservation and Domestication of a Neglected and Endemic Vine
Source: Plants (Basel). 2025 Oct 10;14(20):3126. doi: 10.3390/plants14203126 (PMC12567215; doi:10.3390/plants14203126)
Supplement: Supplementary file 1 [file plants-14-03126-s001.zip › plants-3817970-supplementary/Table S5.pdf]

Table S5. Weight of internal structures (g) of *Lardizabala biternata* fruits. Edible pulp weight (EPW), seeds weight (SdW), peel weight (PeW) and edible pulp plus peel weight [(EP+Pe)W].

Morphological traits	Population	Weight of internal structures (g)				
		**Mean**	**Min**	**Max**	**SD**	**CV (%)**
**Edible pulp weight (g) (EPW)**	**StCr16**	10.8	5.1	17.3	3.3	30.7
	**Vald16**	9.8	5.8	14.6	2.9	29.8
	**Vald18**	11.5	7.5	17.2	3.4	29.6
	**Differences by location (StCr16 vs Vald16)**	ns				
	**Differences by seasons (Vald16 vs Vald18)**	ns				
**Seed weight (g) (SdW)**	**StCr16**	7.6	1.6	11.6	3.1	40.3
	**Vald16**	5.8	3.5	7.9	1.4	24.4
	**Vald18**	9.7	5.7	13.6	3.1	31.9
	**Differences by location (StCr16 vs Vald16)**	*				
	**Differences by seasons (Vald16 vs Vald18)**	***				
**Peel weight (g) (PeW)**	**StCr16**	7.3	4.7	9.8	1.6	21.5
	**Vald16**	4.4	1.7	8.6	2.3	53.4
	**Vald18**	8.4	5.0	10.2	1.9	22.0
	**Differences by location (StCr16 vs Vald16)**	***				
	**Differences by seasons (Vald16 vs Vald18)**	***				
**Edible pulp plus peel weight (g) [(EP+Pe)W]**	**StCr16**	18.0	10.3	26.6	4.2	23.1
	**Vald16**	14.1	8.2	22.9	5.1	35.8
	**Vald18**	19.9	12.5	27.0	5.0	25.4
	**Differences by location (StCr16 vs Vald16)**	*				
	**Differences by seasons (Vald16 vs Vald18)**	***				

Fruits were collected from two locations: Santa Cruz and Valdivia. Data correspond to fruits harvested near Santa Cruz in the 2016 season (StCr16), and in Valdivia during the 2016 (Vald16) and 2018 (Vald18) seasons. The table shows the average of each morphological trait (Mean), minimum (Min) and maximum (Max) weights, standard deviation (SD), and coefficient of variation (CV). Significant differences between locations (StCr16 vs Vald16) and between seasons (Vald16 vs Vald 18) were evaluated using Student's t-test ($P < 0.05$, $P < 0.01$, and $P < 0.001$) and are denoted by *, **, and ***, respectively. "ns" indicates not significant.
