# Supplementary material for: First Phenotypic Characterization of the Edible Fruits of Lardizabala biternata: A Baseline for Conservation and Domestication of a Neglected and Endemic Vine
Source: Plants (Basel). 2025 Oct 10;14(20):3126. doi: 10.3390/plants14203126 (PMC12567215; doi:10.3390/plants14203126)
Supplement: Supplementary file 1 [file plants-14-03126-s001.zip › plants-3817970-supplementary/Table S6.pdf]

Table S6. Eigenvectors of principal component axes from PCA for the morphological traits in Lardizabala biternata fruits.

	Components		
**Trait**	**1**	**2**	**3**
**IFW**	0.324	-0.081	0.062
**FL**	0.287	-0.262	0.008
**FW**	0.259	0.344	0.000
**FH**	0.257	0.354	0.040
**FD**	0.264	0.357	0.020
**FV**	0.323	0.061	0.031
**EPW**	0.291	-0.199	0.046
**SdsW**	0.303	-0.033	-0.174
**PeW**	0.270	0.040	0.317
**(EP+Pe)W**	0.310	-0.100	0.185
**TSdn°**	0.239	-0.413	0.034
**VSdn°**	0.259	-0.341	-0.271
**FSdn°**	-0.109	-0.116	0.866
**ASdW**	0.156	0.445	0.034

IFW: Individual fresh weight; FL: fruit length; FW: fruit width; FH: fruit height; FD: fruit diameter; FV: fruit volume; EPW: edible pulp weight; SdsW: seeds weight; PeW: peel weight; (EP+Pe)W: edible pulp plus peel weight; TSdn°: total seed number per fruit; VSdn°: viable seed number per fruit; FSdn°: non-viable seed number per fruit; and ASdW: average seed weight.
