# Supplementary figures and images for: First Phenotypic Characterization of the Edible Fruits of Lardizabala biternata: A Baseline for Conservation and Domestication of a Neglected and Endemic Vine
Source: Plants (Basel). 2025 Oct 10;14(20):3126. doi: 10.3390/plants14203126 (PMC12567215; doi:10.3390/plants14203126)

**A**

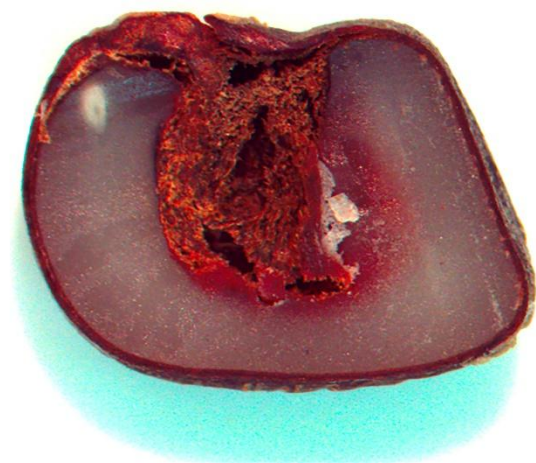

**B**

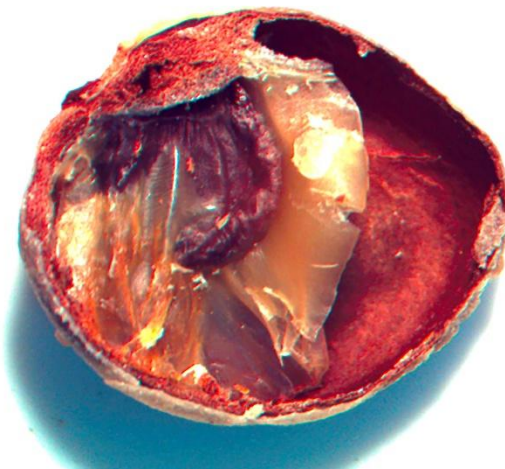

**Figure S2:** Seeds of *Lardizabala biternata* fruits. A) Viable and B) non-viable seeds.

Supplement: Supplementary file 1 [file plants-14-03126-s001.zip › plants-3817970-supplementary/Figure S2.pdf]
